# Transcriptome Sequencing, *De Novo* Assembly and Differential Gene Expression Analysis of the Early Development of *Acipenser baeri*

**DOI:** 10.1371/journal.pone.0137450

**Published:** 2015-09-11

**Authors:** Wei Song, Keji Jiang, Fengying Zhang, Yu Lin, Lingbo Ma

**Affiliations:** 1 Key Laboratory of East China Sea and Oceanic Fishery Resources Exploitation and Utilization, Ministry of Agriculture, Shanghai, China; 2 East China Sea Fisheries Research Institute, Chinese Academy of Fishery Sciences, Shanghai, China; University of Nordland, NORWAY

## Abstract

The molecular mechanisms that drive the development of the endangered fossil fish species *Acipenser baeri* are difficult to study due to the lack of genomic data. Recent advances in sequencing technologies and the reducing cost of sequencing offer exclusive opportunities for exploring important molecular mechanisms underlying specific biological processes. This manuscript describes the large scale sequencing and analyses of mRNA from *Acipenser baeri* collected at five development time points using the Illumina Hiseq2000 platform. The sequencing reads were *de novo* assembled and clustered into 278167 unigenes, of which 57346 (20.62%) had 45837 known homologues proteins in Uniprot protein databases while 11509 proteins matched with at least one sequence of assembled unigenes. The remaining 79.38% of unigenes could stand for non-coding unigenes or unigenes specific to *A*. *baeri*. A number of 43062 unigenes were annotated into functional categories via Gene Ontology (GO) annotation whereas 29526 unigenes were associated with 329 pathways by mapping to KEGG database. Subsequently, 3479 differentially expressed genes were scanned within developmental stages and clustered into 50 gene expression profiles. Genes preferentially expressed at each stage were also identified. Through GO and KEGG pathway enrichment analysis, relevant physiological variations during the early development of *A*. *baeri* could be better cognized. Accordingly, the present study gives insights into the transcriptome profile of the early development of *A*. *baeri*, and the information contained in this large scale transcriptome will provide substantial references for *A*. *baeri* developmental biology and promote its aquaculture research.

## Introduction

Sturgeon is the common name of prehistoric fish species in the family of Acipenseridae belonging to genera such as *Acipenser*, *Huso*, *Scaphirhynchus*, and *Pseudoscaphirhynchusare* [1]. These species have attracted much concern in the scientific field because of their economical and biological relevance and have been classified in the appendix of the endangered animals by the Convention on International Trade in Endangered Species (CITES) of wild fauna and flora [2]. Furthermore, genomics data on sturgeons remain limited despite the fact that they constitute an important archetypal material for studying the origin and evolution of species [3]. Indeed, up to date, the evolutionary relationships among sturgeons have been investigated using anonymous microsatellites and mitochondrial DNA [4]. Only sporadic academic works have focused on gene expression in well-defined biological processes such as phylogenetic distance of *Acipenser baeri* to other fish species [5]. Specifically, comprehensive genomic analyses of the genome of *A*. *baeri*, as well as several species with scientific and economical values, are often hampered by high costs. Moreover, sturgeons possess a polyploid genome with a significant genetic distance to the better scrutinized teleost fishes, rendering comparative genomic approaches extremely difficult [1]. In recent years, transcriptome sequencing has been broadly applied to unearth remarkable information from biological activities, even for several non-model species.

In the case of sturgeons, next-generation sequencing technology has been applied to illustrate gene expression patterns in gonads of *Acipenser fulvescens* and to identify SNPs, sex–determining genes and genes related to xenobiotiques metabolism and their corresponding functions [6, 7]. In addition, other transcriptomes of reproductive tissues from sturgeon have been made available [8, 9]. In a different study targeting microRNA transcriptome and expression assay in *Acipenser schrenckii*, consistent genomic data has been equally provided [3]. Similarly, the largest transcriptomic data concerning the Adriatic sturgeon (*Acipenser naccarii*) has been organized into the public database of *AnaccariiBase* using the RNA-seq technology [8]. Nevertheless, these data are unable to completely explain the development of sturgeons since previous researchers focused on specific organs at unique stages. Moreover, there are few molecular reports on the early development of sturgeons and its relevant regulatory mechanism.

Nevertheless, *A*. *baeri* has tremendous scientific and reproduction benefits and constitutes an important archetypal material for studying the origin of species and evolution. Transcriptome of the early development of *A*. *baeri* will probably give some insights in the comprehension of the regulatory mechanisms of its early development and has important theoretical and practical significance for understanding the development of *A*. *baeri* and other related species. Meanwhile, the study will provide consistent information for diverse biological processes of sturgeons and might be peculiarly valuable for coping with reproduction, growth and health matters in *A*. *baeri*.

Therefore, *de novo assembly* and annotation of transcriptome from RNA sequencing (RNA-seq) were performed from five specimens collected at different developmental stages. We aim to exploit the ensued data for characterizing molecular mechanisms involved in the early development of *A*. *baeri*.

## Materials and Methods

### Fish samples

This study employed specimens of *A*. *baeri* collected at five different developmental stages. Embryos were raised from inseminated eggs provided by commercial suppliers (Hangzhou Qiandaohu Xunlong Sci-tech Development Co. Ltd in China) and kept in a rectangular channel connected to a flow-through fresh water system. Fresh water aquaria were maintained at 18–21°C for erratic periods of time (1 week to several months). The embryos and larvae were staged by developmental time and observations of developmental stages. The developmental stages selected for this study included big yolk plug (T1), wide neural plate formation (T2), canal bud separation (T5), one day old larvae (T9) and eleven days old larvae (T17) stages. The characteristics of each sample are listed in Table 1.

**Table 1:** Characteristics of fish specimens.

Sample Name	Developmental stages	Morphological characteristics
**T1**	Big yolk plug stage (32 hours after fertilization)	The formation of large yolk plug
**T2**	Wide neural plate formation (45 hours after fertilization)	The wide neural plate is obvious and segmented into internal and external parts
**T5**	Canal bud separating stage (65 hours after fertilization)	Formation of the heart primordium and protrusion of tail bud within the mesenchymal rod
**T9**	1 day old larvae	Raised head, increased pigmentation in the middle of eyes; apparition of triangular notch, beginning of gill differentiation; apparition of transparent yolk sac in the ventral surface
**T17**	11 days old larvae	Exuberant larvae feeding, swimming in the bottom, apparition of small granular protuberances between the ventral medial tentacles, apparition of ampullary organ primordia around the eyes, gill cover and ventral surface

### Ethics statements

Experimental protocols adopted were approved by the Review Committee for the Use of Animal Subjects of Shanghai Ocean University. In China, academic research on endangered species is highly encouraged and does not necessitate particular permits. Sodium pentobarbital was used for anesthesia of larval samples, and all efforts were made for minimizing suffering.

### mRNA library construction and sequencing

At specific developmental stages, whole bodies of embryos or anesthetized larvae were collected and immediately placed in liquid nitrogen until RNA extraction. Total RNA was extracted from 3 specimens per developmental stage using Trizol (Invitrogen, CA, USA) according to the manufacturer’s instructions. The quantity and purity of the extracted RNA were analyzed using Bioanalyzer 2100 and RNA 6000 Nano LabChip Kit (Agilent, CA, USA) with RIN number >8.0. RNA extracted from specimens of each development stage were pooled together as one stage-specific sample. Approximately 10 μg of total RNA was employed for Poly (A) mRNA isolation using oligo-dT magnetic beads (Invitrogen). Subsequently, the mRNA was fragmented into small pieces in the presence of divalent cations (fragmentation buffer (Ambion, #AM8740)) at 94°C for 5 min using an ultrasonicator. The RNA fragments were reverse-transcribed into the cDNA library using the mRNA-Seq preparation kit (Illumina, San Diego, USA). The paired-end sequencing (2*100 bp) on an Illumina Hiseq2000 platform was implemented using paired-end libraries with normal insert size of 300±50 bp.

### Sequencing data quality control and de novo assembly

Prior to assembly and mapping, we applied filters for quality control of sequenced reads. Trim Galore software was employed for quality trimming of raw reads and dynamic removal of adapters and low-quality fragments.

*De novo* assembly of reads was realized using high-quality sequences obtained after quality control using the Trinity platform (http://trinityrnaseq.sf.net) including Inchworm, Chrysalis and Butterfly as independent modules [10]. The assembly was realized in four steps: (1) the analysis of lengths of overlapping k-mers of each sequence and storage of frequencies and k-mer lengths of each sequence in a hash table; (2) the correction and quality processing of k-mer data; (3) transcripts assembly sketch using k-mers with high abundance ratios and high frequencies as seed k-mers; (4) extension of seed k-mers from 5’ to 3’ followed by 3’ to 5’ ends in a coverage-guided manner. The assembly of fragments accepted minimum coverage of 2 (2 x) with length ≥ 2*(k—1). The analysis of the assembly of k-mers were done using the De Brujin graph. The optimal structure of transcripts was obtained after the optimization of the length and path of fragments. In sample assembly splicing and merging assembly splicing, we chose k = 25. After this step, we obtained preliminary linear transcripts. Further optimization of transcripts was carried out using the De Brujin graph for splicing. Thereafter, the adjusted De Brujin graph was converted into a weighted sequence graph in which each node represents a transcript sequence rather than the sequence in the form of k-mer. The nodes were weighted on the basis of the number of sequences of each node and, on the basis of lengths of sequences of the node, the coverage and alternative splicing isoforms of complete sequences were determined.

The evaluation of the efficacy of the assembly was done on the basis of the total number of transcripts, transcripts length distribution and N50 calculation. We computed the N50 size according to the threshold of lengths of different transcripts and counted transcripts with lengths greater than or equal to the minimum threshold.

A unigene was defined as the longest transcript among the multitude of transcript isomers. According to the principle of assembly, the optimization of assembly process allowed the obtaining of different transcript isomers (isoform) or paralogs. Each unigene (expressed in prefix comp + digital ID) corresponded to one or numerous transcripts isomers (comp*_c*_Seq*).

### Prediction of ORF or CDS

To discriminate between valid transcript sequences and incorrectly assembled sequences, we used TransDecoder program integrated in Trinity software (log likelihood ratio based on the ratio of coding to noncoding sequences) to extract open reading frames (ORFs) and predict potential protein coding domain sequences (CDS) based on Markov model principle. CDS were translated into amino acid sequences according to the standard codon table in order to obtain potential protein sequences coded by the transcripts.

### Functional annotation of unigenes

To pinpoint conserved protein domains and ascribe putative efficient information to unigenes, we matched translated amino acid sequences stemmed from assembled unigenes to manifold functional databases. Unigene sequences were first aligned against UniProt protein databases (Swissprot or Tremble) using BLASTX algorithm with an E-value cut off of 1E-3. Gene annotation was assigned to unigenes based on the highest BLAST hit. We also mapped unigenes to the Gene Ontology (GO) database using Blast2GO (http://www.blast2go.de) [11]. Annotation via Blast2GO was done by first searching for matches to the Uniprot databases, then mapping the BLAST results to the GO database and finally retrieving GO annotation information corresponding to Uniprot protein sequence number. WEGO software was used for GO functional classification. The KEGG annotation of unigenes were achieved using the online KEGG database (http://www.genome.jp/kegg/). Using the HMM algorithm, we employed the interproscan software (http://www.ebi.ac.uk/InterProScan/) to detect sequence matches and predict protein characteristics, such as function, signal peptide, transmembrane domain characteristics and spiral structure. The search in Interpro included integrated databases such as PRINTS, SMART, Pfam, Coils, SUPERFAMILY, Gene3D, ProSiteProfiles, Hamap, ProSitePatterns, TIGRFAM and PIRSF. We chose final unigenes by comparing results of all of the BLAST and functional domain database estimations and retaining only sequences that disclosed a noteworthy connection to at least one database.

### Data deposition

The sequencing data were deposited in the NCBI Short Read Archive (SRA) database (http://www.ncbi.nlm.nih.gov/sra/) under the accession number SRP053165.

### Unigenes abundance estimation and analysis of differentially expressed genes

To compute abundance estimates of transcripts, the sequenced reads were re-aligned to the assembled transcripts using a script in Trinity [12–14]. Subsequently, the RNA-Seq by Expectation-Maximization (RSEM) package (default parameter Settings) was used to ascribe reads to unigenes or isoforms and to count transcript abundances in FPKM units [13]. For the identification of differentially expressed genes (DEGs), the Bioconductor tool DESeq package (p-value < 0.05) was exerted for pairwise comparison between samples following the protocol described elsewhere [14, 15]. The RSEM values (expected count) were input in DESeq for generating the base mean of each unigene in compared samples. DESeq computed the 'fold change' as the ratio of the base mean (reads number after homogenization) of sample 2 (later stage) to the base mean of sample 1 (earlier stage). The 'fold change' was then log2 transformed (Log_2_(Fold Change)). Under-expressed genes had negative Log_2_ values while over-expressed gene had positive Log_2_ values. For the analysis of significance of DEGs, the q-values were calculated from all unigenes p-values after correction for multiple testing using the Benjamini-Hochberg adjustment procedure. The hierarchical clustering of DEGs were done using Cluster 3.0 and the heat map was visualized by TreeView 1.6 (EisenLab, Stanford University, Stanford, California).

### GO and KEGG enrichment analysis of DEGs

To have an overview on the functions of DEGs, screened differentially expressed unigenes were mapped to terms in GO and KEGG databases for gene functional enrichment analysis. The GO functional enrichment procedure was as follows: the total gene set was considered as a background list and differential genes list as the screened list obtained from the background list. The hyper-geometric test was used to calculate the p-value in order to evaluate the significance of GO-terms of DEGs. The hyper-geometric formula employed for p-value calculation was given as follows:

$$\text{P}=\;\sum_{i=0}^{m-1}\frac{\left(\begin{array}{c} M\\ i \end{array}\right)\left(\begin{array}{c} N-M\\ n-i \end{array}\right)}{\left(\begin{array}{c} M\\ n \end{array}\right)}$$

Note: N stands for the number of genes with GO annotation among all genes/transcripts, n is the number of DEGs in N, M is the number of all genes that are annotated to a certain GO term; and m is the number of DEGs in M.

The formula used to compute the significance of pathways in KEGG enrichment analysis was similar to that used in the GO enrichment analysis. Here, N is the number of genes with a KEGG annotation, n is the number of DEGs in N, M is the number of genes annotated to specific pathways, and m is the number of DEGs in M.

FDRs (false discovery rate) were obtained after correction for multiple testing of the p-value using Benjamini and Hochberg method. Pathways with a p-value< 0.05 were defined as significantly enriched pathways after correction for multiple testing.

### Expression profile and GO enrichment analysis of clusters of DEGs

The determination of expression patterns of DEGs through developmental stages was achieved with clusters generated by the Short Time-series Expression Miner (STEM) [16, 17]. This algorithm employs exclusive methods for clustering, comparing, and visualizing data and provides useful and statistically rigorous biological explanations of short time series data owing to its integration with the Gene Ontology. GO enrichment for differentially co-expressed unigenes were carried out using the hypergeometric distribution algorithm.

## Results

### Sequencing data analysis and *de novo* assembly

The statistics of the original and pre-processed sequences were shown in Table 2. The Illumina Hiseq2000 paired-end sequencing (2*100 bp) of cDNA obtained from specimens corresponding to the five developmental stages of *A*. *baeri* produced 64109484, 64708472, 79586582, 88487368 and 85889646 reads respectively for T1,T2, T5, T9 and T17 covering 6.41, 6,47, 7.96, 8.85 and 8.59 Gb of sequences. After a thorough quality control and filtering, a total of 64039846 (6.37), 64635214 (6.43), 79514258 (7.92 Gb), 88418042 (8.80 Gb) and 85824746 (8.55 Gb) clean reads were obtained from T1, T2, T5, T9 and T17, respectively. The average lengths of clean reads were 99.5 for T1 and T2, and 99.6 for T5, T9 and T17.

**Table 2:** Statistical results of raw and preprocessed sequences.

Sample Name	T1	T2	T5	T9	T17
**Raw reads**	64109484	64708472	79586582	88487368	85889646
**Raw Gb**	6410948400	6470847200	7958658200	8848736800	8588964600
**Clean reads**	64039846	64635214	79514258	88418042	85824746
**Clean Gb**	6372389027	6433659221	7918179598	8803558655	8545473625
**Average length**	99.5	99.5	99.6	99.6	99.6
**Isoforms**	136941	183065	172644	238500	285089
**Unigenes**	81448	112382	99031	150759	195458

High quality clean reads were employed for *de novo* assembly using trinity software. The assembly generated dissimilar quantities of isoforms for each sample. Specifically, 136941, 183065, 172644, 238500 and 285089 isoforms were obtained respectively for T1, T2, T5, T9 and T17 stages. After the elimination of redundant transcripts, a total of 278167 unigenes were generated from the five samples. Samples T1, T2, T5, T9 and T17 produced 81450, 112382, 99031, 150759 and 195458 unigenes with lengths ranging from 201 to 28205 bp. The N50 statistics of the assembly results was displayed in lengths (S1 File). The calculated N50 value (N50 value = 745) of the assembly results combined with the range of transcript lengths showed that the assembly could normally reflects most of the structure of transcripts. The length distribution of unigenes in each samples was depicted in S1 Fig. The results showed that unigenes with lengths lower than 5000 bp were the most abundant in each library. The huge number of unigenes resulted from the assembly (278167 unigenes) was probably due to a very deep sequencing identifying a mass of low level transcription. This was confirmed in subsequent steps of gene expression analysis using RSEM software where transcripts with low abundance (FPKM<1) were detected. In addition, this was explainable by the fact that the present study covers the whole transcriptome of embryos and larvae and could be able to detect some rare transcripts. These results demonstrated the reliability of Illumina paired-end sequencing and de novo assembly. In-depth functional analysis was obtained in the subsequent steps, and further confirmed the reliability of sequencing and splicing analysis.

### Functional annotation of unigenes

To identify the putative function of *A*. *baeri* transcripts, all the sequences were blasted against the reference proteins available in NCBI Uniprot protein databases using BLASTX with an E-value cutoff of 1E-3. The best blast matches are reported in S2 File. A total of 57346 unigenes (20.62%) had significant hits to Uniprot databases corresponding to 45837 unique known proteins and 11509 homologous orthology clusters in Uniprot protein databases. The remaining 79.38% of unigenes probably represent non-protein coding genes, UTRs or transcripts from *A*. *baeri*-specific genes which were too dissimilar to be annotated by homology search with the adopted E-value cutoff. We proceeded to BLASTX analysis in an attempt to determine species with which the top hits were generated (S3 File). The Blast2 best hit with taxa in the Uniprot databases showed the hits of *A*. *baeri*’s transcripts with 1312 distinct species. As expected, a commendable portion of our sequences had their top BLASTX hits with functional transcripts in fish species such as *Latimeria chalumnae* (5940 transcripts), *Danio rerio* (4943 transcripts), *Helobdella robusta* (4591 transcripts) and *Oreochromis niloticus* (2325 transcripts) and in minor extent to other vertebrate species.

The GO functional classification was done by the alignment of unigene sequences with Uniprot database and the retrieval of GO terms associated to Uniprot protein sequences number. The GO annotation results are reported in S4 File. A total of 43062 unigenes (15.5%) were assigned at least one GO term in the categories of “biological process”, “molecular function” and “cellular component”.

In the KEGG database (S5 File), 29526 unigenes (10.61%) were annotated into 329 pathways. “Metabolic pathways” (ko01100, 2010 transcripts) was the most represented pathway and was followed by “biosynthesis of secondary metabolites” (ko01110, 642 transcripts) and “microbial metabolism in diverse environments” (ko01120, 417 transcripts). In the pathway class of signal transduction, “PI3K-Akt signaling pathway” (ko04151, 347 transcripts) was the most represented. Other pathways related to diseases and translation were similarly extant. Overall, 27773 unigenes were annotated in both GO and KEGG databases.

Interproscan was used to identify conserved domains, or functional units, within the protein query sequences. A total of 16735 unigenes (6.02%) were annotated in Interproscan and generated 6751 domains (S6 File). The statistics (S7 File) showed that “zinc finger, C2H2” was the most predominant conserved domain (count = 3392) followed by “WD40 repeat” (count = 2312) and “zinc finger, C2H2-like” (count = 1799). These annotations bestow a precious resource for exploring specific processes, functions and pathways during research on *A*. *baeri*.

### Expression level distribution and identification of DEGs

The expression level of each transcript was determined using FPKM method as described in material and methods section. The distribution of the expression level of unigenes in each sample is depicted in S2 Fig while the correlation between the expression level and lengths of the transcripts was plotted in S3 Fig. For respective T1, T2, T5, T9 and T17 samples, 81448, 112382, 99031, 150759 and 195458 transcripts were found expressed with FPKM values ranging from 0.01–18809.06. Genes with FPKM < 1 were considered as lowly expressed genes. After filtering low level expression transcripts, 35841, 45258, 36026, 50844 and 77935 transcripts were obtained as actively expressed transcripts for T1, T2, T5, T9 and T17, respectively. The mapping results also showed that 38071 transcripts were expressed at all stages whereas 5424, 7673, 4381, 16819 and 68020 genes were specifically expressed in respective T1 (FPKM ≤ 190), T2 (FPKM ≤ 33), T5 (FPKM ≤114), T9 (FPKM ≤ 319) and T17 (FPKM ≤ 3365.06).

DESeq software was used for screening DEGs between pairwise compared samples. A total of 3479 genes were identified as DEGs within the five developmental stages. The DEseq output files were compiled in S8 File. The statistics of DEGs among samples was summarized in Table 3. Gene annotation revealed that 2447 of these genes were annotated as species conserved genes while the remained were not annotated and could be probably considered as DEGs specific to *A*. *baeri*.

**Table 3:** Statistics of differentially expressed genes among samples.

**Sample**	**Total number of DEGs**	**Number of up-regulated DEGs**	**Number of down-regulated DEGs**	**Number of DEGs specific to earlier stage**	**number of DEGs specific to later stage**
**T1> T2**	150	78	32	1	39
**T1>T5**	601	331	94	9	167
**T1>T9**	675	325	163	27	160
**T1>T17**	1171	525	205	49	392
**T2>T5**	117	98	1	0	18
**T2>T9**	313	194	58	16	45
**T2>T17**	791	402	92	38	259
**T5>T9**	44	23	15	3	3
**T5>T17**	381	144	52	13	172
**T9>T17**	313	83	13	3	214

### GO and KEGG pathway enrichment analysis of DEGs

GO and KEGG pathway enrichment analysis was performed for the functional annotation and classification of DEGs using the list of DEGs screened from the integral set of genes.

**Fig 1:**
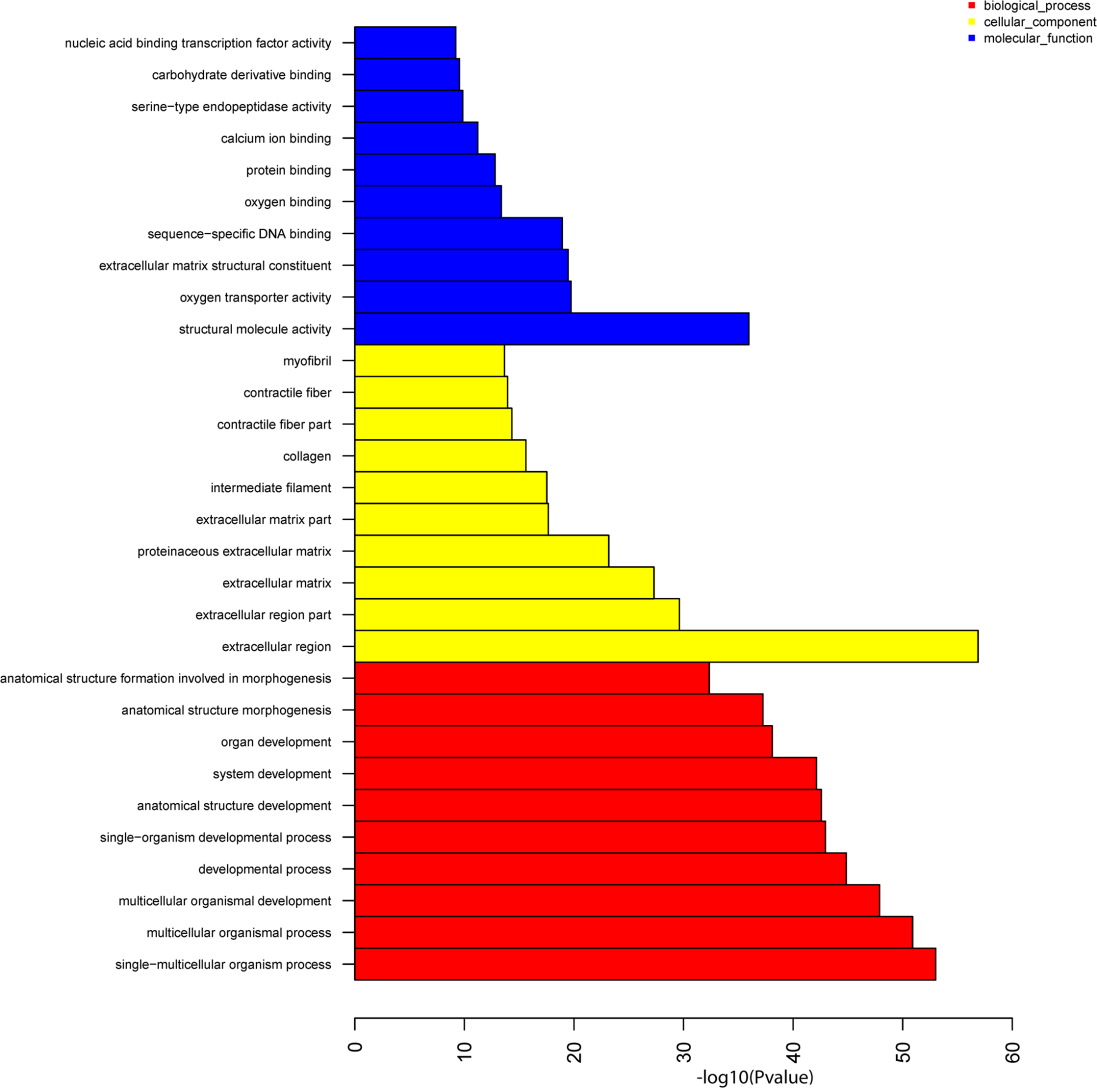
Histogram of the most significantly enriched GO terms obtained from the GO enrichment analysis of all DEGs. The significance of each GO term was estimated based on the p-value (p-value < 0.05) and the FDRs (FDR ≤ 1.82E-01); blue color denotes “molecular functions”, yellow represents the “cellular component” and red the “biological process” categories.

The GO enrichment analysis of DEGs across the five developmental stages led to 1857 significant (p-value < 0.05, FDR ≤ 1.82E-01) GO terms that were clustered into “biological process”, “cellular component” and “molecular function” categories. Fig 1 shows the histogram of the top 10 significantly enriched GO terms in each GO class. All significantly enriched GO terms were summarized in S9 File.

In the category of “biological process”, single-multicellular organism process (487 unigenes), multicellular organismal process (492 unigenes) and multicellular organismal development (402 unigenes) were the greatest significant dynamic processes. For cellular component category, 274 unigenes were in charge of extracellular region, 152 unigenes controlled extracellular region part and 111 unigenes were involved in extracellular matrix showing that extracellular activities are fundamental during the development of *A*. *baeri*. The predominant molecular functions encoded by DEGs included structural molecule activity (184 unigenes), oxygen transporter activity (25 unigenes) and extracellular matrix structural constituent (34 unigenes) as the topmost significantly enriched GO terms. This suggested that gene regulation is crucial for achieving various physiological functions associated to the development of *A*. *baeri*.

**Fig 2:**
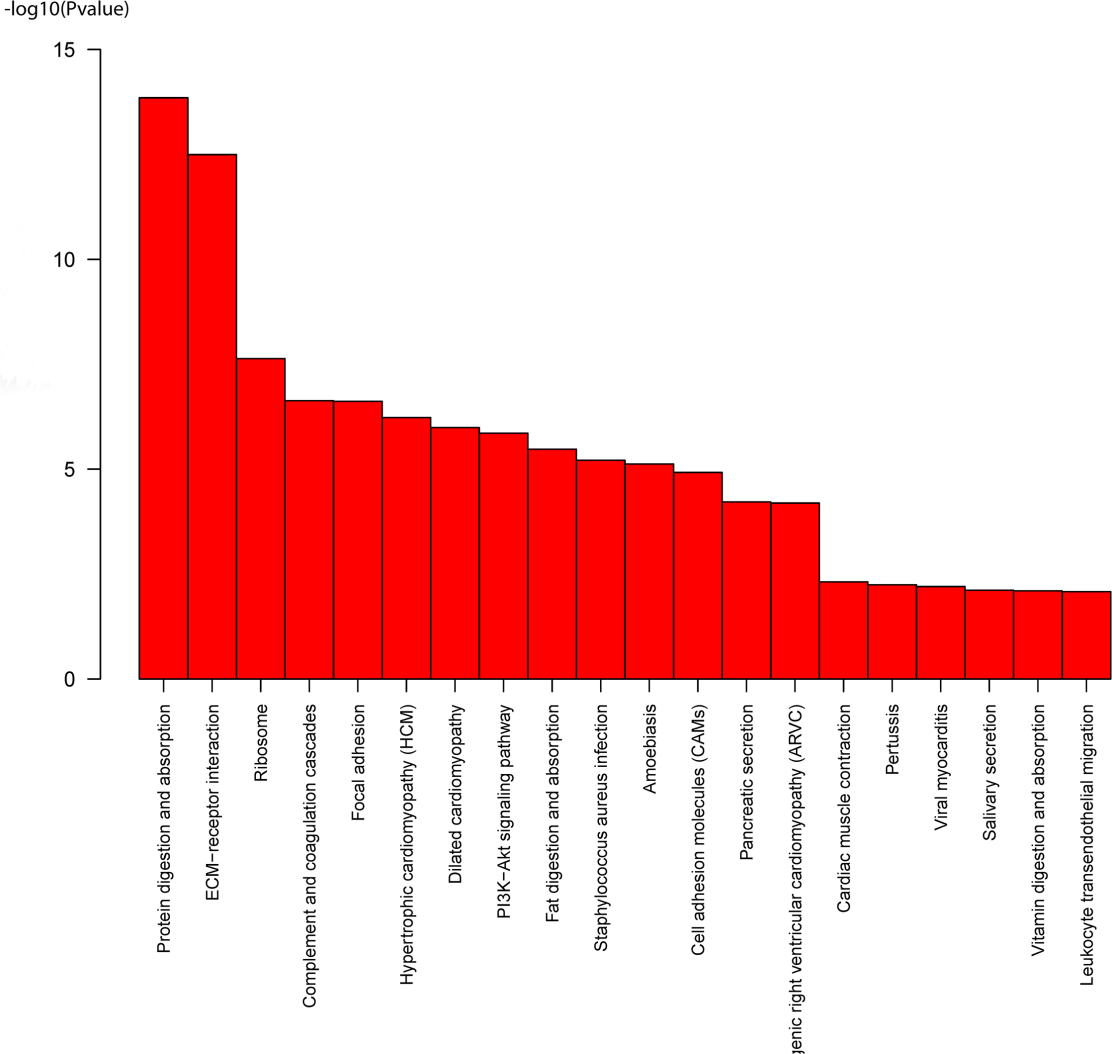
Histogram of the most significantly enriched KO terms obtained from the KEGG enrichment analysis of DEGs. The significance of each pathway was estimated based on the p-value (p-value < 0.05) and the FDRs (FDR ≤ 1.82E-01)

In order to uncover pathways mostly in play during *A*. *baeri*’s early ontology, the KEGG pathways enrichment of DEGs was performed. Fig 2 shows the top 20 significantly enriched KO pathways while all significantly enriched KO terms were summarized in S10 File. The result revealed significant enrichment for 49 metabolic pathways (p-value < 0.05, FDR ≤ 1.82E-01) with protein digestion and absorption (32 unigenes), ECM-receptor interaction (28 unigenes), ribosome (58 unigenes) and complement and coagulation cascades (19 unigenes) as the most prevalent pathways. These observations disclosed the vital implications of signaling molecules and interaction, digestive system, cell communication and signal transduction in developmental processes of *A*. *baeri*. In total, 18 classes of metabolic pathways were recorded and included development, cell growth and death, cancer related, immune system, immune diseases, bacterial and parasitic diseases, signal transduction and endocrine system metabolic pathways. The above pathways might play important roles in the development of *A*. *baeri*.

### Dynamic expression profiles of DEGs

**Fig 3:**
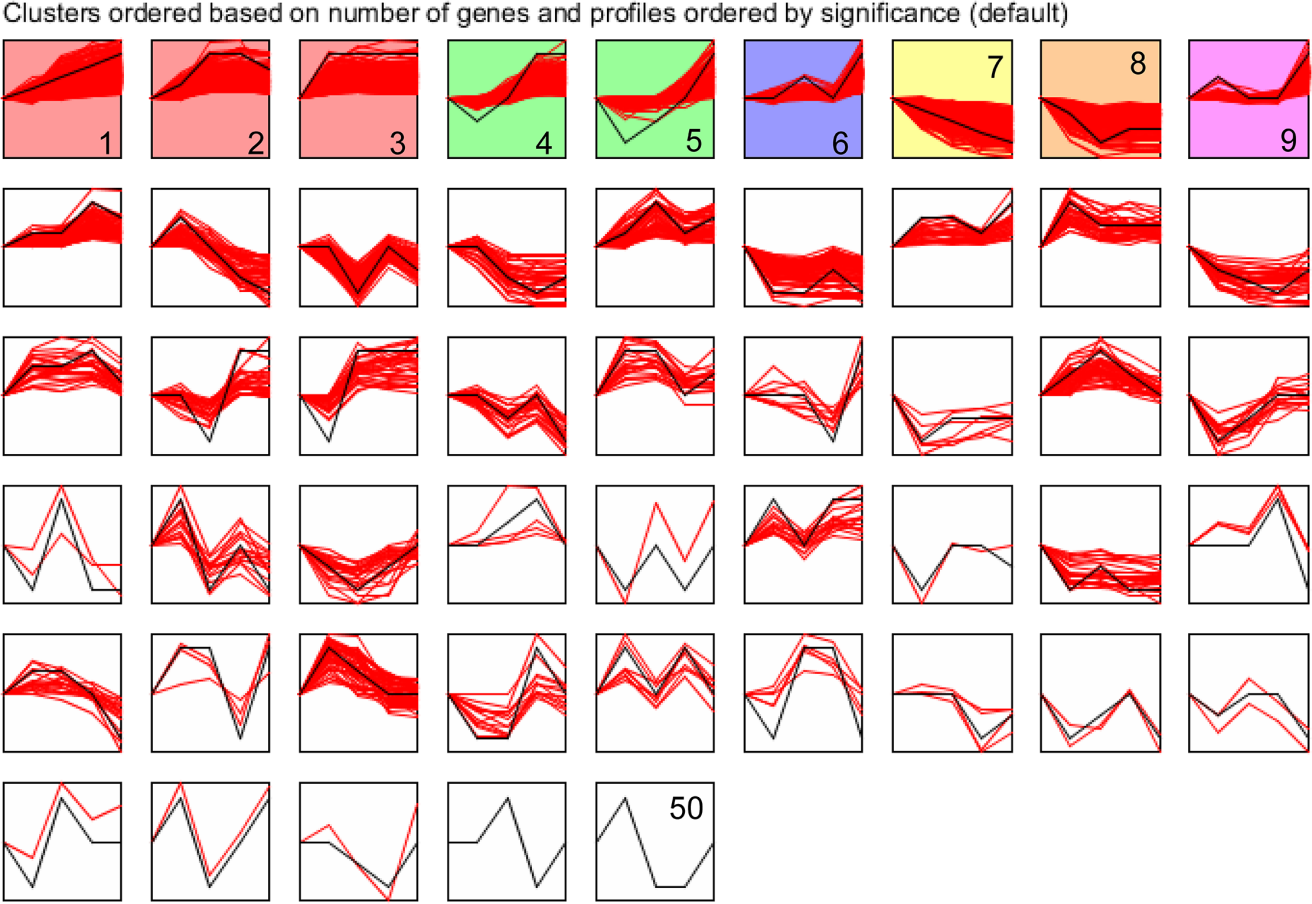
Expression profile and clusters of DEGs obtained from the STEM clustering. Numbers indicated clusters or profiles. Clusters were ordered according to the number of genes while profiles were ordered according to significance. Significantly different profiles were represented by different background colors.

To survey dynamic expression patterns of DEGs during development, we employed STEM software to classify all the DEGs according to their abundance changes. The DEGs were classified into 50 clusters according to their expression patterns. Each cluster covered a certain number of co-expressed genes with a well-defined expression pattern. Clusters were ordered according to the number of genes while profiles were ordered according to significance (Fig 3). Nine significant expression profiles (profiles 1 to 9) were identified. As shown in Fig 3, significantly different profiles were represented by different background colors. Respectively, 833, 276, 275, 177, 141, 241, 233, 226 and 180 co-expressed DEGs were identified for profiles 1 to 9. In order to gain a glimpse of the biological functions of these 9 clusters of genes, we implemented GO enrichment analysis. The result of GO enrichment analysis of these groups of genes presenting their biological functions is summarized in S11 File. We found that genes co-expressed in profiles 1 to 9 separately participated in 3229, 792, 538, 513, 223, 204, 820, 494 and 193 biological functions (GO terms). Profiles 1 and 7 presented interesting features because, on one hand, their DEGs were enriched for high number of GO terms, and on the other hand, these groups of genes had opposite expression trends. Indeed, the expression levels of DEGs of profile 1 (0, 1, 2, 3, 4) were constantly increased across chronological development stages while the opposite tendency was found for DEGs of profile 7 (0, -1, -2, -3, -4). These two clusters of genes with opposed expression tendencies endowed very significant data about gene functions in the development of *A*.*baeri*. The GO functional analysis (S11 File) showed that genes of profile 1 were significantly involved in 794 biological processes of which, the highly significant included single-multicellular organism process, muscle system process and contraction, extracellular matrix organization, anatomical structure formation involved in morphogenesis and multiple processes related to development. At the cellular component level, these genes encoded for 91 components which were significantly represented by extracellular region and contractile structures (GO:0044449, GO:0043292, GO:0030017, GO:0030016). The upper significant molecular functions (160 in total) were extracellular matrix structural constituent and calcium ion binding. The 233 genes assigned to profile 7 were majorly involved in embryo development (GO:0009790) and embryonic morphogenesis (GO:0048598) and were expressed for cellular components including nucleus (GO:0005634) and its related modules such as chromatin (GO:0000785), chromosomal part (GO:0044427), chromosome (GO:0005694) and nucleosome (GO:0000786). Their molecular functions were associated with DNA and nuclear bindings.

Equally involved in diverse developmental processes, the cluster of 275 DEGS assigned to profile 3 (0, 1, 1, 1, 1) displayed a characteristic expression pattern since they were statically expressed during the development process after their upregulation at T2. These genes were enriched for molecular functions including sequence-specific DNA binding, nucleic acid binding transcription factor activity and sequence-specific DNA binding transcription factor activity. These genes were likely involved in the regulation of diverse biological processes.

Detailed information of the GO enrichment of the six remaining significant expression profiles are contained in S11 File. The other 41 profiles presented dissimilar gene clusters and genes numbers with characteristic expression changes at specific stages of development (Fig 3).

### DEGs preferentially expressed at stages

**Fig 4:**
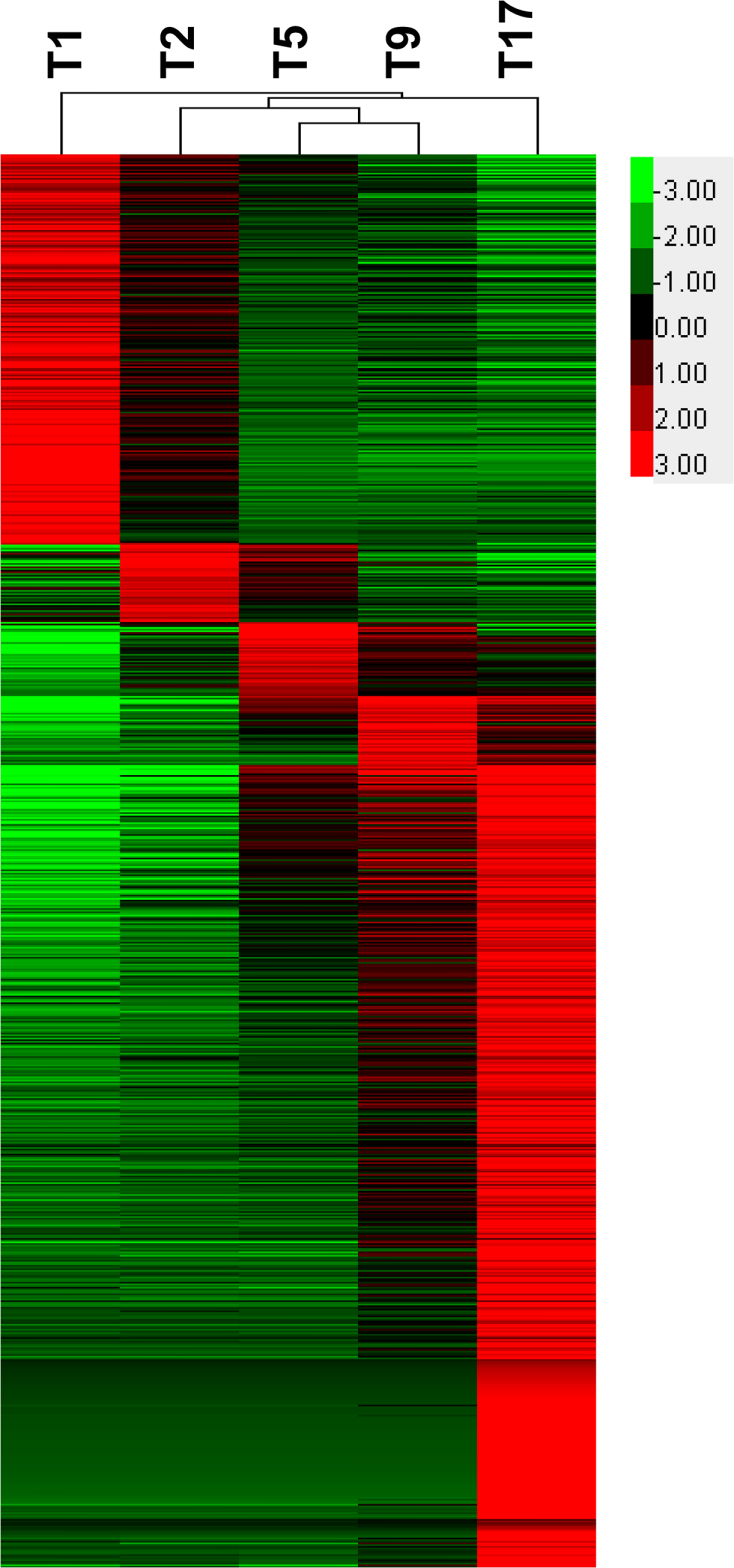
Heat map of expression patterns and hierarchical clustering of DEGs at different stages. Only DEGs of a given stage presenting significant expression differences by comparison with other stages were considered as genes preferentially expressed at this stage.

To identify DEGs preferentially expressed at each stage, we screened the cohort of DEGs that was highly expressed in a stage and had statistically significant changes in expression compared with other stages. Fig 4 represents the heat map obtained from the hierarchical clustering of these genes. Different amount of genes were preferentially expressed at each stage as presented in S12 File, with the largest number of detected stage-preferential DEGs found at T17 (954 DEGs) followed by T1 (464 DEGs). For T2, T5 and T9, we respectively identified 93, 88 and 82 stage-preferential DEGs. The set of DEGs detected as preferentially expressed at a developmental stage were analyzed for GO and KEGG enrichment (S13 File) in order to determine physiological functions and metabolic pathways highly associated with that stage.

The top 20 of DEGs identified as preferentially up-regulated at T1 included genes such as *hLAMP-1*, *Hmga1*, *ccnb1*, *SOX32*, *g3bp1*, *zgc*:*101772*, *GTSF1*, *cldna*, *LOC101064479*, *nanog*, *srsf1b*, *srsf1*, *ZPAX*, *zgc*:*66432*, *PANDA_011403*, *LOC100694009*, *LOC100356102*, *LOC733726*, *ccne1* and *mkrn4* genes. The GO enrichment analysis of DEGs preferentially expressed at T1 showed their implications in 957 physiological functions with embryo development (GO:0009790) as the most significant GO term in the category of “biological process”, nucleus as the significant cellular component and DNA binding as the most active molecular function. We recorded 25 metabolic pathways through KEGG pathway enrichment analysis with cell cycle as the most active pathway, followed by genes involved in modules well denoted by melanogenesis, Wnt signaling pathway, Hedgehog signaling pathway, Hippo signaling pathway and dorso-ventral axis formation.

The biological processes predominantly activated at T2 included response to copper ion, endocrine pancreas development, response to ethanol, growth and response to oxygen-containing compound. The molecular functions were significantly represented by transcription related regulatory activities. In total, T2-preferential DEGs were enriched for 405 GO terms while the KEGG enrichment led to the identification of 6 metabolic pathways including folate biosynthesis, aminobenzoate degradation and steroid biosynthesis. The highly expressed of these genes were characterized as *BRAFLDRAFT_125880*, *cldn30c*, *alcam*, *APOD*, *UY3_06366*, *ada*, *cldn6*.*2*, *LOC798445*, *nos3*, *ENTPD1*, *LOC101075370*, *TREES_T100011293*, *DLEU7*, *LOC100709469* and *Cdx1*.

The 88 DEGs preferentially expressed at T5 were enriched for 340 GO terms and were specially involved in the biological processes related to circulatory and muscular functions including heart contraction, heart process, regulation of heart contraction, blood circulation, circulatory system process and muscle contraction. The TNF signaling pathway, leukocyte trans-endothelial migration and ascorbate, aldarate metabolism carbohydrate metabolism and Bladder cancer were the 4 significant enriched KEGG pathways. The most highly expressed genes included *TMSB4X*, *RL31*, *MAP1LC3C*, *mlc2*, *Tnni3*, *tll1*, *ldha*, *zgc*:*162989*, *LOC101164642*, *PKHF1*, *zgc*:*162509*, *TREES_T100021613*, *TNNT2*, *cpn1*, *MLRA*, *FKBP7*, *HAO1*, *D623_10031084*, *PLLP* and *fhl2a* genes.

Similarly, the 82 DEGS preferentially expressed at T9 regulated diverse biological functions (481 GO terms) including multicellular organismal catabolic process, triglyceride metabolic process, acylglycerol metabolic process and multiple immune related functions. These genes had molecular functions like peptidase related activity, chemokine and G-protein coupled receptor binding and phosphoenolpyruvate carboxykinase (GTP) activity. Among the 11 KO terms, digestion metabolic pathways and immune system related pathways were the most abundant and corroborated with the GO enrichment analysis result. These genes included *AFP4*, *UY3_18160*, *hbb2*, *Anapl_03192*, *DDX42*, *TTR*, *tnni1*.*2*, *cebpb*, *pgc*, *LOC446273*, *PCK1*, *thbs1b*, *alb2*, *LOC100696099*, *LOC100547249*, *LOC100699459*, *col4a5*, *mmp* and *LOC100691625*.

Finally, the huge number of genes specifically over-expressed at T17 were specialized in translation, oxygen transport, gas transport, electron transport chain and various respiratory processes. The most enriched molecular functions included structural molecule activity, structural constituent of ribosome and oxygen transporter activity. Ribosome, protein digestion and absorption, ECM-receptor interaction and pancreatic secretion were the most significant pathways obtained from the KEGG enrichment analysis of these genes. Signaling pathways such as PPAR signaling pathway, PI3K-Akt signaling pathway and adipocytokine signaling pathway were the regulatory pathways found at this stage. Genes highly expressed at this stage were *Angj1*, *GSTENG00015950001*, *myl1*, *LOC101170382*, *myhc4*, *COL1A3*, *TRPII*, *ACTC1*, *COL1A1*, *ckm*, *Gkn2*, *pgc-A*, *tnnt3b*, *tnni2*, *col1a2*, *Tryp2*, *LOC100556781*, *CSA*.*11056*, *LOC100477257*, *AMBP*, *COI*, *MUC5B*, *CEL2A*, *serpina1*, *LGALS4*, *LOC101171131 a*nd *LOC100564029*.

## Discussion

The early development of *A*. *baeri* include embryonic and larval stages and embroils intense changes, which provides a unique opportunity for studying relevant biological processes [18]. Former research on *A*. *baeri* development focused on histological analysis of digestive tract and eye [19, 20]. The early ontogeny and allometric growth patterns of the species were also investigated [18]. A better comprehension of *A*. *baeri* development would be particularly valuable for reproduction control, the long-term sustainability of *A*. *baeri* and other sturgeon species management and aquaculture. However, the shortage of genomic data concerning this species is a critical drawback for meeting the demand. Fortunately, the expansion of sequencing technologies has sped up the generation of transcriptomics data even for scarce species.

In order to understand the molecular mechanism implicated in the development of *A*. *baeri*, transcriptome sequencing of this species was performed at different stages. Different libraries of unigenes were obtained from the *de novo* assembly of the sequencing data stemming from *A*. *baeri*. Different number of isoform transcript was obtained for each sample. After the elimination of redundant transcripts, a total of 278167 unigenes were generated by merging unigenes obtained from the five samples. The number of transcripts obtained was largely over the 11187 transcripts obtained from the RNA-seq of specimens originated from the early development of Zebrafish [21]. The huge number of unigenes obtained was explainable, on one hand, by the fact that the RNA-seq technology allowed the detection of low level transcription genes, and on the other hand because the transcriptome was obtained from whole bodies of embryos and larvae of *A*. *baeri*. The present study makes it possible to overview the developmental transcriptome of *A*. *baeri*. The transcriptome data obtained here is the first report of high-throughput gene expression data for the early development of *A*. *baeri*. With the 278167 unigenes assembled, the data described characterize substantial progress in sturgeon genetics. In combination with the previous transcriptome sequencing data of sturgeon species [3, 6, 8], the genomic data obtained herein will potentially and significantly contribute in aquaculture and conservation research. Portions of the assembly unigenes were annotated in available databases, thus revealing the efficacy of the assembly and allowing the identification of functional unigenes.

The bioinformatics analysis of sequencing data showed dramatic alterations of gene expression with a total of 3529 DEGs screened from the five developmental stages. The GO enrichment analysis showed that the DEGs were significantly involved in developmental processes and molecular functions including structural molecule activity, oxygen transporter activity, extracellular matrix structural constituent, sequence-specific DNA binding, oxygen binding and protein binding. These results revealed the importance of respiratory and gene regulation functions during the early development of *A*. *baeri* and corroborated with the findings on O_2_-chemoreceptive pathways in the larval development of zebrafish [22]. The KEGG enrichment showed that protein adsorption and digestion was the most significant pathways in play during the early ontogeny of *A*. *baeri*. This could be useful information for fish feeding since the result corroborated with the findings of other researchers who stated that different dietary protein concentrations dissimilarly impacted on the physiology of *A*. *baeri* and suggested different biotechnological functions [23]. ECM-receptor interaction and ribosome were also significantly enriched, thus displaying the key role of translation and protein interaction in the development of *A*. *baeri*. Conventionally, the extracellular matrix (ECM) offers signaling clues that control cellular activities and coordinate functions in tissue development and differentiation, and deregulations of cell–ECM connections can be a factor of developmental and immune related disorders [24]. The development of metazoan species implicates synchronized cellular multiplication, growth and differentiation that lead to systematized tissues and necessitates fitted control of gene expression facilitated by manifold transcriptional and translational regulation mechanisms involving ribosome [25, 26].

The STEM clustering of DEGs revealed 50 expression patterns organized in clusters. The most significant profile (833 genes with constant up-regulation during development) were involved in a multitude of biological processes dominated by muscle related processes, morphogenesis, system development and so on. The result was consistent with previous findings stipulating that morphogenesis and differentiation are important biological processes in the early development of fish [18]. The cluster of genes with expression profile characterized by constant down-regulation within the five developmental stages were those in charge of embryonic development and morphogenesis. The expression pattern of genes of this cluster revealed high transcriptional activity of genes involved in the embryo development at early stages of development and the shrinking of their regulation over the development course.

Different amount of genes were preferentially expressed at specific stages and revealed dramatic changes among stages. The highest number of up-regulated genes was recorded at T17 followed by T1. Low numbers of upregulated genes were found in T2, T5 and T9. This suggested that T1 and T17 are critical stages in the early ontogeny of *A*. *baeri*. The identification of genes preferentially expressed at stages shed light into the molecular mechanisms occurring at each developmental stages and provided better understanding for gene expression changes during the development of *A*. *baeri*.

## Conclusions

In the present study, we successfully generated the transcriptome profile of the early ontogeny of *A*. *baeri*. The study provided an approach of molecular mechanisms in the regulation of the early development of *A*. *baeri* and the functions of DEGs. The results will contribute greatly to sturgeons’ research and aquaculture. We intend to exploit obtained *in silico* results for further molecular applications on *A*. *baeri*.

## Supporting Information

S1 Fig Length distribution of unigenes assembled from each developmental stage. (TIF)

S2 Fig Distribution of the expression level of unigenes in each sample. (TIF)

S3 Fig The correlation between the expression level of transcripts and the transcripts length. (TIF)

S1 File N50 statistics of the sequencing data. (XLS)

S2 File Results file of the best matches of unigenes obtained from the alignment against Uniprot database. (XLS)

S3 File Statistics of hits of *A*. *baeri*’s sequences to species in the Uniprot database. (XLS)

S4 File Unigenes GO annotation results file. (ZIP)

S5 File Unigenes KEGG pathway annotation results file. (XLS)

S6 File Unigenes interpro annotation results file. (XLS)

S7 File Interproscan domain statistics. (XLS)

S8 File DESeq output files for pairwise comparison of samples for identification of DEGs. (ZIP)

S9 File Significantly enriched GO-terms obtained from the GO enrichment analysis of DEGs. (XLS)

S10 File Significantly enriched pathways obtained from the KEGG pathway enrichment analysis. (XLS)

S11 File GO enrichment analysis of sets of genes assigned to the nine most significant expression profiles. (XLS)

S12 File Lists of genes preferentially expressed at each development stage. (XLSX)

S13 File Significant GO terms and pathways obtained from GO enrichment analysis and KEGG pathway enrichment of each set of stage specific genes. (XLS)

S14 File GO enrichment analysis of genes used to build the regulatory network in terms of molecular function (MF), cellular component (CP) and biological processes (BP). (XLSX)
