# Supplementary figures and images for: Transcriptome Sequencing, De Novo Assembly and Differential Gene Expression Analysis of the Early Development of Acipenser baeri
Source: PLoS One. 2015 Sep 11;10(9):e0137450. doi: 10.1371/journal.pone.0137450 (PMC4567377; doi:10.1371/journal.pone.0137450)

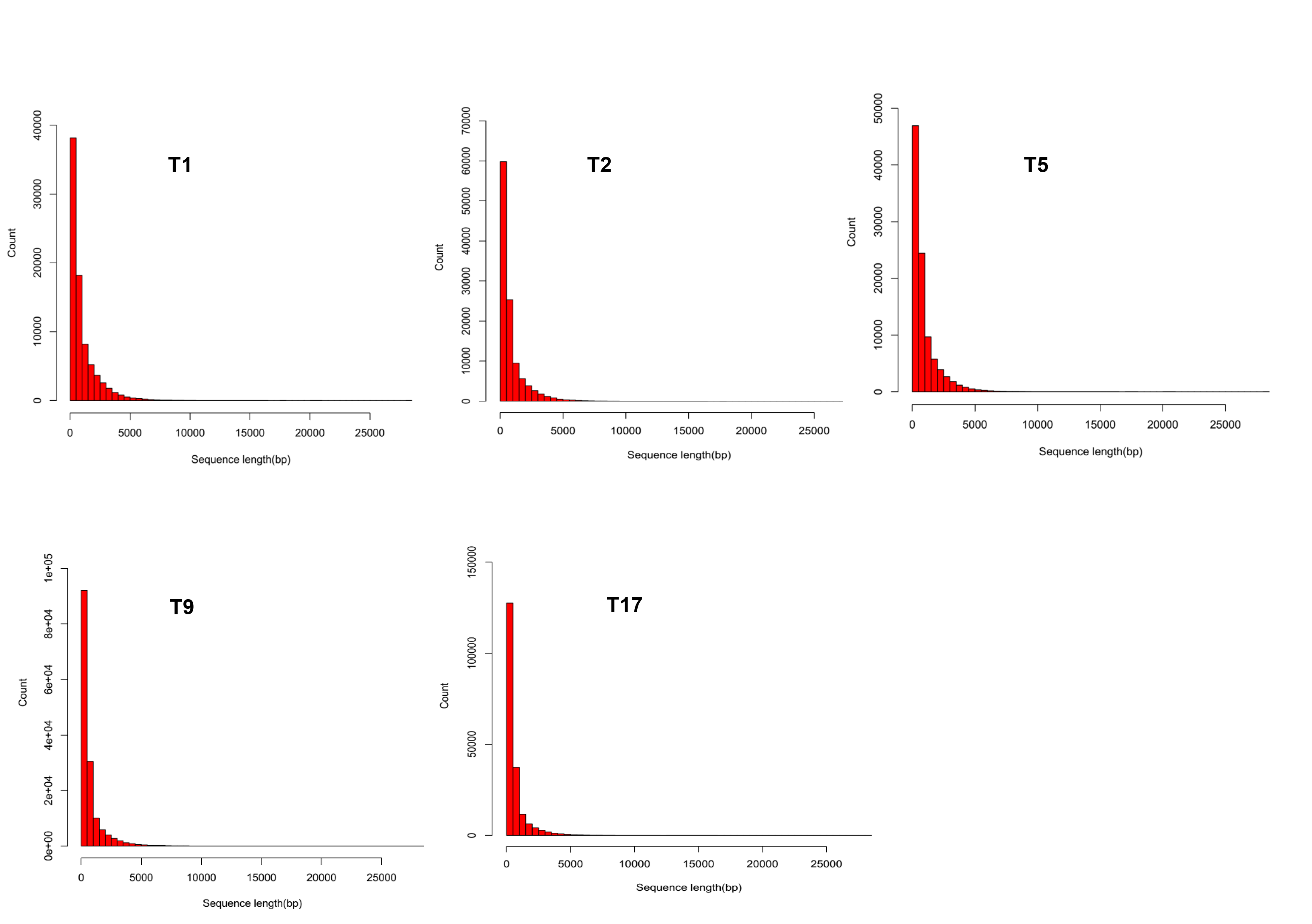

Supplement: S1 Fig — (TIF) [file pone.0137450.s001.tif]

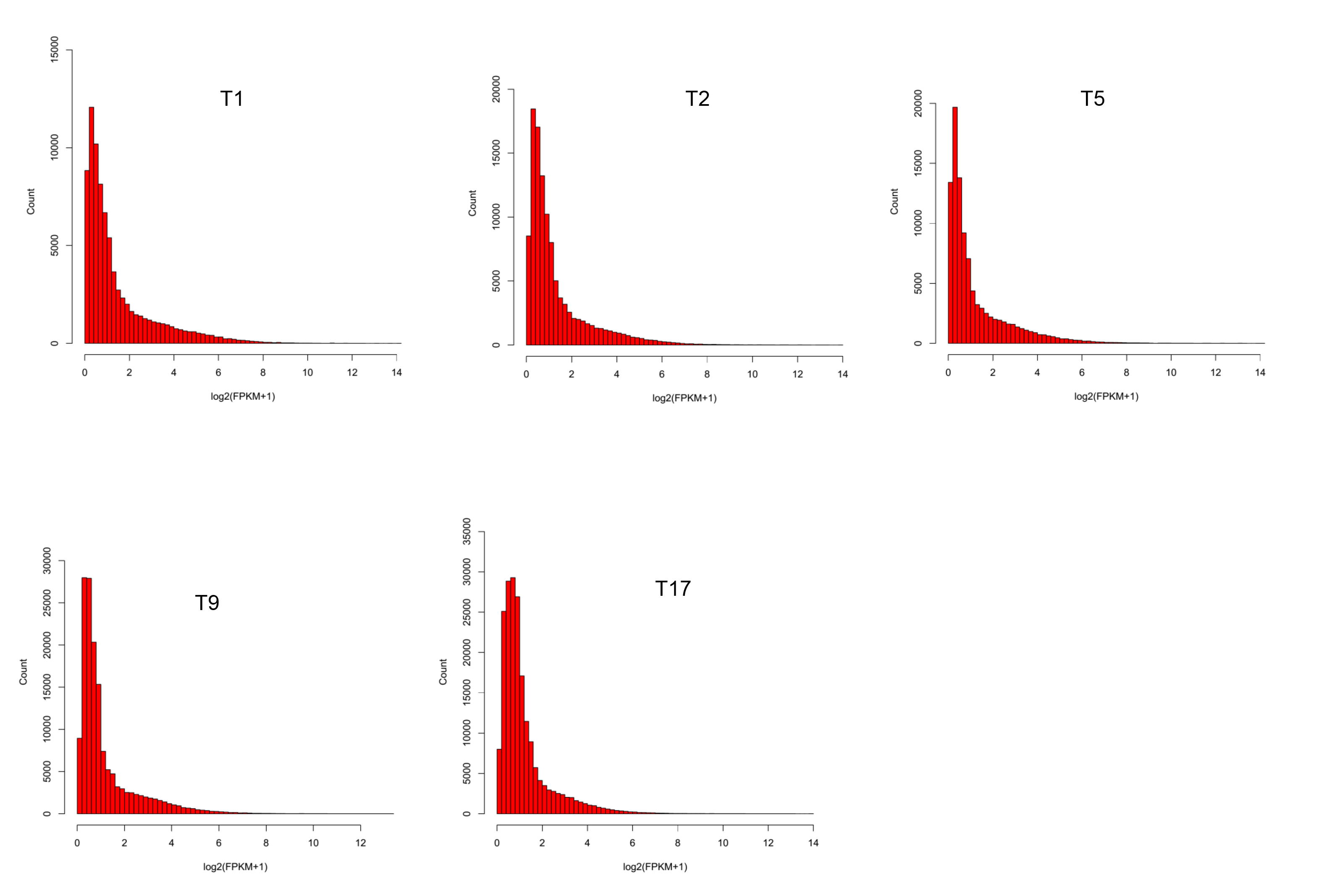

Supplement: S2 Fig — (TIF) [file pone.0137450.s002.tif]

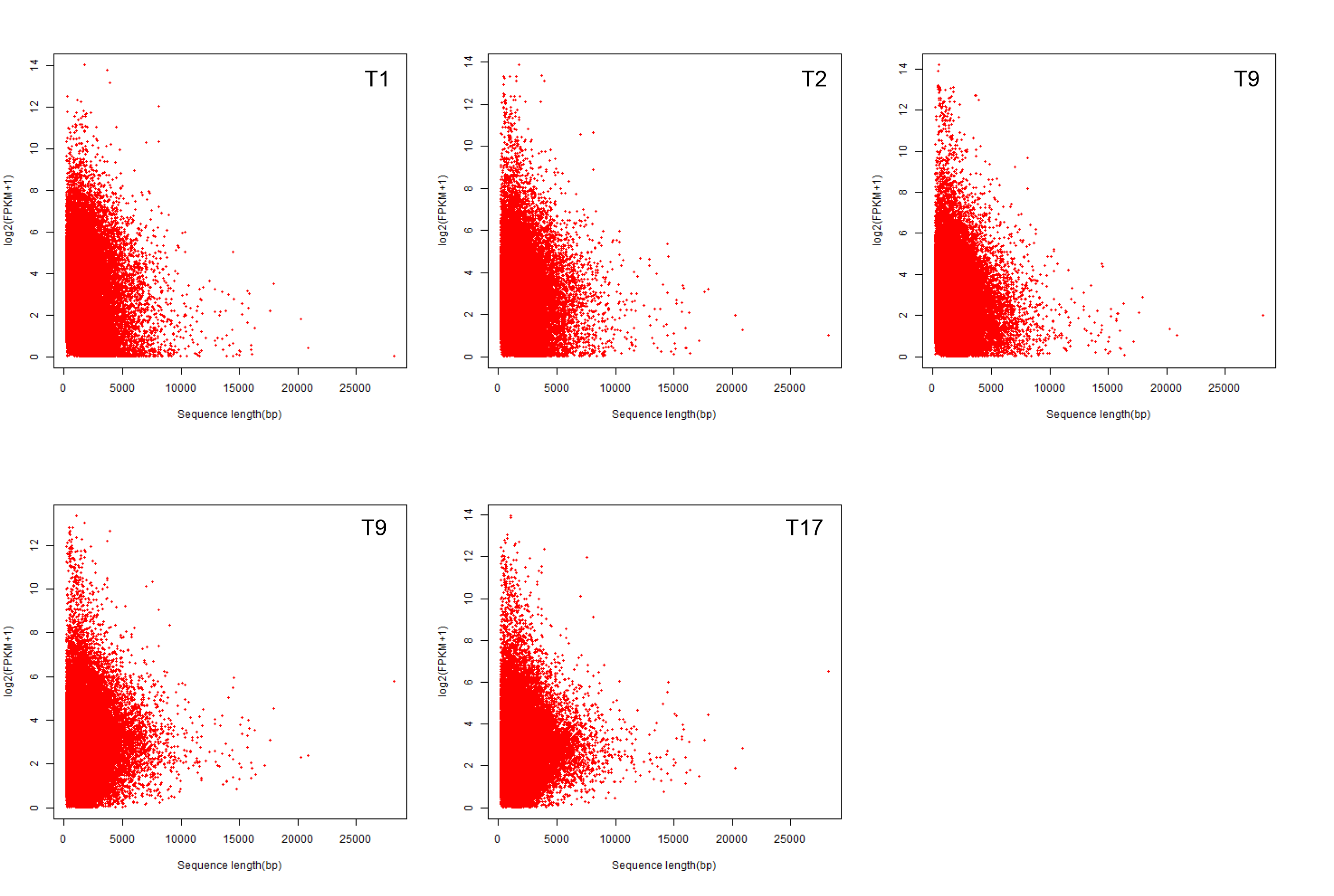

Supplement: S3 Fig — (TIF) [file pone.0137450.s003.tif]
